# CT and MRI Features of Pediatric-Aged Colloid Cysts: Report of Two Cases

**DOI:** 10.1155/2017/2467085

**Published:** 2017-01-31

**Authors:** Hidayatullah Hamidi, Fazel Rahman Faizi, Najibullah Rasouly, Mer Mahmood Shah Hoshang

**Affiliations:** French Medical Institute for Children (FMIC), Kabul, Afghanistan

## Abstract

A 10-year-old boy with the history of headache, anorexia, and vomiting was referred to our department to undergo a brain CT scan. CT images demonstrated a well-defined, rounded, hyperdense lesion at the level of the foramen of Monro causing moderate dilatation of the lateral ventricles. An 11-year-old girl with a long history of a headache was also referred to undergoing a brain MRI. MR images demonstrated a well-defined round abnormal signal intensity lesion at the level of the foramen of Monro causing moderate dilatation of lateral ventricles. The findings from imaging perspective were consistent with the colloid cyst of the third ventricle. Therefore, the diagnosis of the colloid cyst was made.

## 1. Introduction

Colloid cysts are benign intracranial neoplasms constituting 1% of CNS tumors. They may be totally asymptomatic or may manifest with symptoms of raised intracranial pressure. The symptoms may be intermittent, self-limiting, and nonspecifically apparent when the foramen of Monro is blocked temporarily by pendulous movement of the cyst or may be acute and severe presenting with acute hydrocephalus, brain herniation, and sudden death. They usually appear as hyperdense lesions on CT; however, they can manifest as isodense or hypodense lesions as well. The appearance of the lesion on MRI is variable on different sequences and is dependent on cholesterol and protein contents of the cyst.

## 2. Case Presentation

### 2.1. Case  1

**Figure 1:**
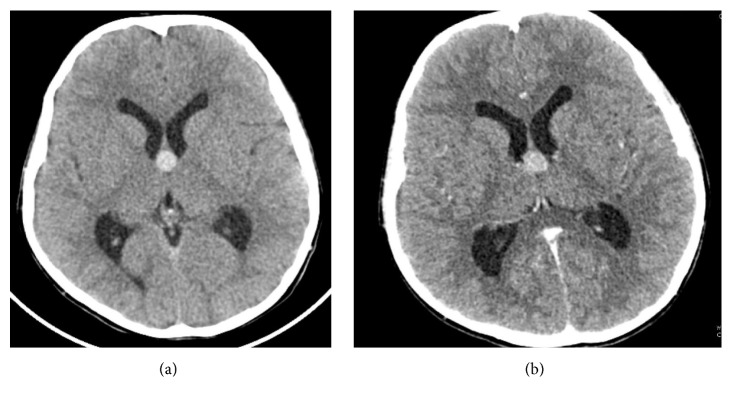
(a) Nonenhanced axial section at the level of the foramen of Monro: a well-defined, round, hyperdense lesion causing mild prominence of bilateral lateral ventricles. (b) Contrast enhanced image shows no significant enhancement in the lesion.

A 10-year-old boy with the history of headache, anorexia, and vomiting was referred to Radiology Department of French Medical Institute for Children to undergo a brain CT scan. The CT scan was performed with 128 slices' Siemens scanner. Precontrast images demonstrated a well-defined, rounded, hyperdense lesion at the level of the foramen of Monro causing moderate dilatation of the lateral ventricles. No specks of calcification were appreciated in the cyst. No significant enhancement appeared after intravenous contrast injection (Figures 1(a) and 1(b)).

### 2.2. Case  2

Interestingly, an 11-year-old girl with a long history of headache was referred to undergoing a brain MRI. The MRI was performed with 1.5 Tesla Siemens machine.

**Figure 2:**
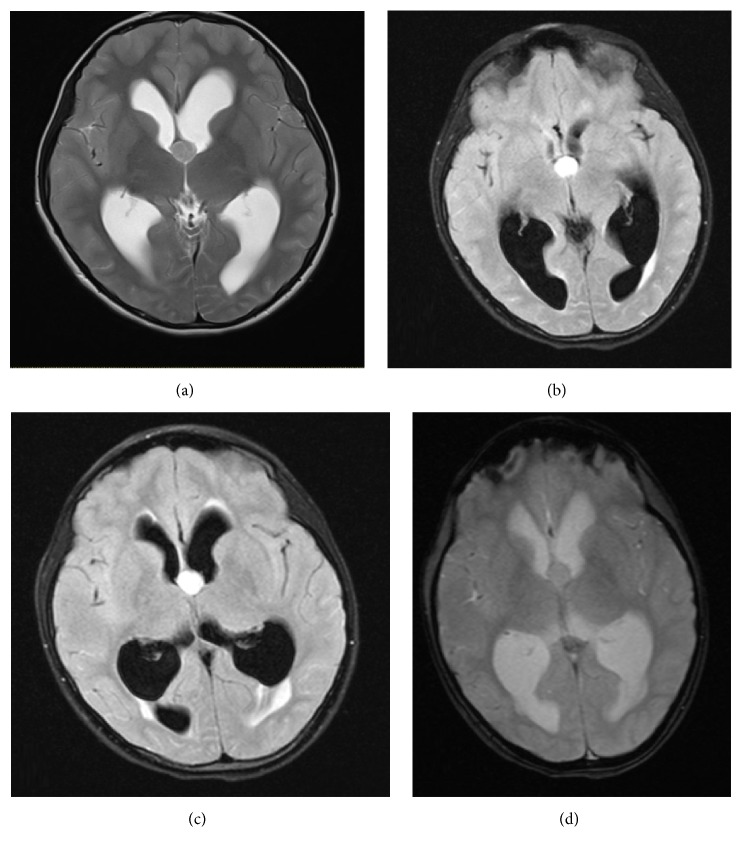
(a) Axial T2WI images: a well-defined, round, isointense lesion to the gray matter at the level of the foramen of Monro causing moderate acute hydrocephalus. (b, c) FlAIR and T1WI: the lesion is hyperintense relative to brain parenchyma. (d) T2^*∗*^ GRE images: no drop of signal to indicate intralesional hemorrhage.

**Figure 3:**
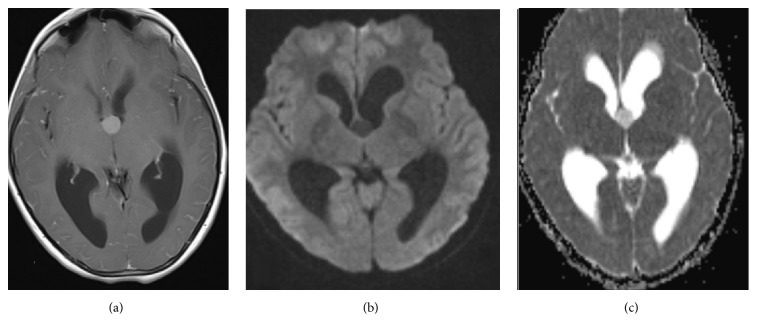
(a) TIWI + C: subtle enhancement after intravenous gadolinium injection. (b & c) No elevated diffusion pattern.

A well-defined rounded abnormal signal intensity lesion was visualized at the foramen of Monro resulting in moderate acute hydrocephalus. The lesion was isointense to gray matter on T2WI and hyperintense on T1WI and FLAIR images. No drop of the signal was visualized in T2^*∗*^ GRE sequence to suggest intralesional hemorrhage. No diffusion restriction was noted. Subtle enhancement of the lesion was seen on postcontrast images (Figures 2 and 3).

The first case did not receive any surgical treatment and, after ten months of follow-up, he claimed that the symptoms have diminished. The second case was lost of follow-up.

## 3. Discussion

Colloid cysts are benign congenital tumors of the brain located in the anterosuperior part of the third ventricle comprising 1% of CNS tumors and occur in three individuals per million per year [1–4]. The age of onset is between 10 and 68 years of life with 68% occurring in third and fourth decades of life [5]. In our case, both patients were too young, in their early second decade of life.

In 1965, Shuangshoti et al. suggested that these cysts originate from neuroepithelium, like ependyma and choroid plexus, hence the term neuroepithelial cyst [6]; however, in 1992, Tsuchida et al. offered a nonneuroepithelial origin of colloid cyst, indicating its similarity to respiratory mucosa of the trachea and sphenoid sinus by using immunohistochemical techniques [7]. It has been postulated that colloid cysts and Rathke cleft cysts may present the same lesion in different locations [1, 8].

Colloid cysts are histologically benign and may be entirely asymptomatic, with no clinical symptom, and may be discovered incidentally. However, they may obstruct the foramen of Monro, raise intracranial pressure, and cause acute hydrocephalus [1, 2, 9].

### 3.1. Clinical Perspective

In symptomatic patients, headache is the presenting sign occurring in 68–100% of cases. It lasts seconds to minutes and initiates, is exacerbated, or terminates by a change in position [1, 3, 10]. Gait disturbances (47%) and short-term memory disturbances (37%) are the two other common symptoms; meanwhile, papilledema (47%) and ataxia (32%) are the most common signs [3]. The symptoms may be intermittent presenting when the foramen of Monro is obstructed by episodic pendulous movement of the cyst, though some may present with acute hydrocephalus, brain herniation, and sudden death [1, 3]. Acute hemorrhage within the cyst is a rare life-threatening condition due to the rapid development of obstructive hydrocephalus or exacerbating preexisting hydrocephalus which requires immediate diagnosis and surgical intervention [11, 12].

### 3.2. Imaging Workup

CT and MRI are both useful for diagnosis of the colloid cysts. On CT, they appear as round or oval shaped hyperdense lesions in the rostral aspect of the third ventricle; they may appear as isodense and hypodense lesions as well [1, 3]. On MRI, about 50% of colloid cysts are hyperintense on T1-weighted images and the rest are either isointense or hypointense with respect to the brain; on T2-weighted images, most colloid cysts are hypointense [1]. Isointense lesions may be difficult to identify on MRI; therefore, CT images are more useful [1, 3].

In a study performed by Sener NR, he claimed that colloid cyst had hypointense signal on DWI (on the *b* = 1000 sec/mm) which were apparently higher than cerebral parenchyma and lower than CSF. The diffusion MR imaging features of the colloid cyst are consistent with an elevated diffusion pattern [13].

Preoperative detection of the rare entity of hemorrhage within the cyst remains a challenge since these cysts usually appear hyperdense on CT images and hyperintense on T1W images. However, in isodense cysts, hyperdense areas may be visible inside the lesion representing acute hemorrhage on CT images [11, 12].

### 3.3. Treatment and Prognosis

Definitive treatment of colloid cyst is surgical excision, through an open craniotomy, endoscopy, or stereotactic aspiration of the cyst contents. However, treatment of asymptomatic patients is dependent upon a number of factors like the lesion size, the presence of hydrocephalus, the age of the patient, and medical conditions [3].

Surgical excision of colloid cyst is challenging due to its deep midline anatomical position [5].

## 4. Conclusion

Colloid cysts of the third ventricle are rare intracranial neoplasm and can affect young individuals. As surgical treatment of colloid cyst is challenging due to its deep midline anatomical position, it is better to treat young patients conservatively. One of our cases revealed diminishing symptoms after ten months of follow-up.

## Additional Points

*Place*. The work is carried out in French Medical University for Children (FMIC), Kabul, Afghanistan.
